# Self-Esteem and Occupational Factors as Predictors of the Incidence of Anxiety and Depression among Healthcare Workers during the COVID-19 Pandemic in Latvia

**DOI:** 10.3390/ijerph21010065

**Published:** 2024-01-07

**Authors:** Laura Valaine, Māra Grēve, Maksims Zolovs, Gunta Ancāne, Artūrs Utināns, Ģirts Briģis

**Affiliations:** 1 Department of Psychosomatic Medicine and Psychotherapy, Riga Stradiņš University, LV-1046 Riga, Latvia; gunta.ancane@rsu.lv (G.A.); arturs.utinans@rsu.lv (A.U.); 2 Statistics Unit, Riga Stradiņš University, LV-1048 Riga, Latvia; mara.greve@rsu.lv (M.G.); maksims.zolovs@rsu.lv (M.Z.); 3 Institute of Life Sciences and Technology, Daugavpils University, LV-5401 Daugavpils, Latvia; 4 Department of Public Health and Epidemiology, Riga Stradiņš University, LV-1010 Riga, Latvia; girts.brigis@rsu.lv

**Keywords:** self-esteem, occupational factors, depression, anxiety, healthcare workers, COVID-19

## Abstract

The prevalence of depression and anxiety among healthcare workers (HCWs) during the COVID-19 pandemic is high. The aim of the study is to identify the importance of self-esteem and occupational factors in association with the incidence of depression and anxiety among HCWs through a longitudinal cohort study during the COVID-19 pandemic in Latvia. Participants received seven questionnaires during the COVID-19 pandemic on demographic parameters, work-related information, and contact with COVID-19 patients, and three standardized questionnaires that evaluated symptoms of anxiety (GAD-7), depression (PHQ-9), and self-esteem (Rosenberg’s self-esteem scale). The Generalized Linear Mixed Model (GLMM) was used to identify factors associated with the incidence of depression and anxiety among HCWs. A total of 322 participants were included in the data analysis for depression and 352 for anxiety. HCWs with low self-esteem were 83% more likely to experience depression and 76% more likely to experience anxiety. Working at a general practitioner practice is associated with twice the risk of developing depression and anxiety. A 31% increase in the odds of depression is observed among HCWs with direct contact with COVID-19 patients. The organizational and government levels must look for opportunities to facilitate the mental health of HCWs to ensure better-quality healthcare.

## 1. Introduction

The meta-analysis of cross-sectional studies shows a high prevalence of depression and anxiety among healthcare workers (HCWs) during the COVID-19 pandemic. Globally, anxiety is more prevalent than depression among HCWs [1,2].

The systematic review that included 18 longitudinal studies published until 2022 from 11 countries claims that the prevalence of depression and anxiety among HCWs increases during COVID-19 outbreaks and decreases after outbreaks but remains at a level of concern [3]. However, longitudinal studies from Italy published in 2023 show controversial data. A study with 407 participants, who were surveyed three times over one year, reports an increase in the rate of stress and depression; however, it remains at a subclinical level [4]. Another study with two-level screening carried out over one year, involving a baseline sample of 990 participants and a follow-up sample of 310 participants, shows a decrease in psychiatric symptoms due to the gradual remission of post-traumatic symptoms and reduced workload pressure [5].

Occupational factors such as workplace, work unit, working hours, occupation, availability of personal protective equipment, and contact with COVID-19 patients were well studied in the HCW population during the COVID-19 pandemic. The results of a meta-analysis representing 458,754 hospital HCWs report that working in high-risk units and being in direct contact with COVID-19 patients are associated with a higher risk of insomnia, sleep disorders, depression, anxiety, and post-traumatic stress disorder (PTSD) [3]. Being a nurse is a risk factor for anxiety, depression, and insomnia [4,5,6].

A person’s sex is known to be an important factor in their mental health. Cross-sectional studies conducted in the early stages of the COVID-19 pandemic report that female HCWs experience anxiety and depression more frequently [3], but a longitudinal study from Italy with repeated measurements carried out between 2020 and 2022 reports opposing findings: that the importance of sex decreased during the pandemic [5].

The impact of personality factors, such as self-esteem, on poor mental health outcomes was not widely studied during the COVID-19 pandemic. It is known that previous mental health status, coping strategies, perceived social support, relationships, and living status are associated with mental health outcomes among HCWs during the COVID-19 pandemic [4]. A cross-sectional study from Qatar reports higher resilience among nurses with higher self-esteem and self-compassion during the COVID-19 pandemic [7]. Some studies have been conducted on the importance of self-esteem in the burnout of HCWs before the COVID-19 pandemic. The results of a cross-sectional study with 306 participants carried out in India indicate an association between low self-esteem, stress, and burnout [8]. A cross-sectional study from Spain involving 719 HCWs measured the association between social support, cognitive and affective empathy, self-esteem, and burnout. The results showed that low self-esteem is the main factor in high burnout rates [9]. Self-esteem is an important factor for professional skills and choice of workplace. Nurses with high self-esteem want to change their workplace to a better one if the stress at their current workplace is too high [10]. Some studies in the general population report an association between self-esteem and symptoms of depression and anxiety. Low self-esteem contributes to depression—there is less erosion of self-esteem during depression; the relation of low self-esteem with anxiety is more symmetrical. However, longitudinal studies are still needed to explore whether low self-esteem is a predictor of poor mental health [11,12].

There is a gap related to limited research on the association between self-esteem, depression, and anxiety among HCWs during the COVID-19 pandemic and some controversial data on the importance of occupational and sociodemographic factors. Caramello et al. highlight the importance of data selection time: in their longitudinal study, follow-up was carried out in the summer when the COVID-19 infection rates were lower and the mental health of the HCWs had improved [13]. During the first COVID-19 wave, Latvia had good results in controlling COVID-19 infection among European Union countries; however, rates of depression and anxiety among HCWs were not far behind those countries that have been hit hard by COVID-19; during the second COVID-19 wave, Latvia was characterized by high mortality rates of COVID-19 [14,15,16]. Latvia is among the countries with a shortage of physicians and nurses, and the pandemic highlighted the lack of HCWs [17]. In our study, we want to observe HCWs who did not have symptoms of depression or anxiety at the start of the COVID-19 pandemic and to determine which factors are associated with new cases of depression and anxiety among HCWs during the COVID-19 pandemic. The aim of the study is to identify the association of self-esteem and occupational factors with the incidence of depression and anxiety among HCWs through a longitudinal cohort study during the COVID-19 pandemic in Latvia.

## 2. Materials and Methods

### 2.1. Ethics

This study was approved on 20 April 2020 by the Research Ethics Committee of Riga Stradiņš University, Latvia, protocol number 6-1/04/1. To ensure a longitudinal study, research participants needed to provide an email address and phone number in the first questionnaire, which was used to contact them about further surveying. The questionnaire did not ask for names, surnames, or other personal information, but, in some cases, the email addresses provided did contain the person’s name and surname. 

The research respects the protection and confidentiality of participants’ data under Regulation (EU) 2016/679 of the European Parliament and the Council (27 April 2016) on the protection of natural persons regarding the processing of personal data and the free movement of such data and repeals Directive 95/46/EC (General Data Protection Regulation) and other applicable laws and regulations. The personal data—phone number and email address—were intended solely for communication purposes in the further stages of the study (for delivering further questionnaires). The anonymity and confidentiality of the research participants were ensured within the requirements of the research design. After the research was completed, the data were stored in anonymized form. The contact information specified in the questionnaire was permanently deleted.

Participation in the study was voluntary. Not participating in the study did not affect the performance of medical care. Participants received informed consent forms and information on the purpose of the study and process. Electronic or written informed consent for participation in the study was obtained from each participant. Participants could withdraw from the survey at any time without providing any justification.

### 2.2. Study Design and Sampling

This study is part of a web-based longitudinal observational study to examine the mental health of HCWs during the COVID-19 pandemic in Latvia. Participants received seven questionnaires during the COVID-19 pandemic at fixed time intervals: T0—from 28 April 2020 to 2 June 2020; T1—3 months after T0; T2—6 months after T0; T3—10 months after T0; T4—13 months after T0; T5—16 months after T0; and T6—19 months after T0. Participants were selected using a non-probability sampling approach. Physicians, physician assistants, nurses, and other HCWs (nursing assistants, physiotherapists, dental technicians, medical students, etc.) were recruited in Latvia from the intensive care and patient admission departments of three university hospitals and fourteen regional hospitals, as well as from state emergency medical services (SEMS), general practitioner (GP) practices, and the Riga Stradiņš University Institute of Stomatology (Stomatology). 

The HCWs were recruited by the authors of the study or heads of departments or institutions on-site or by sending an invitation by email, depending on the internal rules of the institution on conducting research during the COVID-19 pandemic. 

The first questionnaire (T0) was delivered in two formats: paper and the REDCap web-based format. The format depended on the internal rules of the specific institution based on restrictions on personal contact due to COVID-19. The next six questionnaires (T1–T6) were sent electronically to participants who agreed to continue participating in the study and provided an email address. The format was a self-reported questionnaire designed via the REDCap website.

### 2.3. Study Sample and Follow-Up

T0 resulted in answers from 864 HCWs. A total of 84.1% of the respondents were women; the median age was 40 (IQR 29.0–54.0); a total of 41.5% were physicians; and 45.5% were nurses or physician assistants. A total of 44.7% worked at hospitals and 22.7% at GP practices. The prevalence of depression and anxiety was 24.8% and 17.2%, respectively. The results related to the T0 evaluation have been published elsewhere [16]. For the longitudinal study, 542 HCWs were excluded from the data analysis for depression symptoms and 512 HCWs from the data analysis for anxiety symptoms because they did not meet the inclusion criteria. Respondents were analyzed from T0 until (1) incidence of depression or anxiety (PHQ-9 ≥ 10; GAD-7 ≥ 10); or (2) last participation in the study without symptoms of depression or anxiety (PHQ-9 < 10 in all interviews; GAD-7 < 10 in all interviews).

### 2.4. Inclusion Criteria

To assess the incidence of depression and anxiety among HCWs during the COVID-19 pandemic in Latvia, three inclusion criteria were established: (1) the HCW worked as an HCW at the previously mentioned healthcare facilities at the time of the study; (2) the HCW did not have symptoms of depression or anxiety (PHQ-9 < 10; GAD < 10) at the time of the first interview (T0); (3) to observe the dynamics of the results of the PHQ-9 and GAD-7 scores, the HCW participated in at least two interviews.

### 2.5. Measurement Tools

Participants reported their demographic parameters, work-related information, and contact with COVID-19 patients and completed three standardized questionnaires that evaluated symptoms of anxiety (GAD-7), depression (PHQ-9), and self-esteem (Rosenberg’s self-esteem scale) in T0–T6.

Each questionnaire included individual characteristics based on similar studies, such as sex (male/female), age, education (≤undergraduate or ≥graduate), occupation (physician, physician assistant, nurse, other), work experience in years, workplace (hospital, SEMS, GP practice, Stomatology), working hours in the last week, and direct contact with patients with a suspected or confirmed case of COVID-19 at the time of the interview.

Self-esteem was assessed using the Rosenberg self-esteem scale (RSS), which measures global self-worth by positive and negative feelings about oneself and consists of 10 questions measured on the Likert scale. The total score range is 10–40. The questionnaire results were classified as categorical variables: low-level (10–25), medium-level (26–29), and high-level (30–40) self-esteem [18,19]. An adapted version was used in Latvian, Cronbach’s alpha 0.84 [20].

Current mental status was assessed using assessment tools in the Latvian language. A validated Latvian version of the nine-item Patient Health Questionnaire (PHQ-9; range, 0–27) was used to assess the symptoms of depression, Cronbach’s alpha 0.84. The results of the questionnaire were categorized as a dichotomous variable. The cut-off score for clinically significant depression was 10 [21,22].

A validated Latvian version of the seven-item Generalised Anxiety Disorder (GAD-7) scale (range 0–21) was used to assess anxiety symptoms, Cronbach’s alpha 0.87 [23,24,25]. The results of the questionnaire were categorized as a dichotomous variable. The cut-off score for clinically significant anxiety was 10 [26].

### 2.6. Data Analysis

This study analyzed the association of self-esteem, demographic factors, and occupational factors measured in T0 with the incidence of depression and anxiety during the COVID-19 pandemic.

The incidence density was calculated using the MedCalc calculator [27]. Person-months were calculated as the time from T0 until (1) the first period when depression or anxiety (PHQ-9 ≥ 10; GAD-7 ≥ 10) was observed; or (2) the last known period without symptoms of depression or anxiety (PHQ-9 < 10 in all interviews; GAD-7 < 10 in all interviews). If respondents did not participate in all consecutive periods, the missing periods were treated as showing no change compared to the previous known period. The number of depression and anxiety events and person-months for the calculation of incidence density were obtained using IBM SPSS Statistics (Version 29.0.0.0).

As this observation study is replicated across time, introducing a random variable, the Generalized Linear Mixed Model (GLMM), was used to identify factors associated with the incidence of depression and anxiety among HCWs. GLMMs are an extension of the linear mixed model, and they are particularly useful when dealing with non-normal distributions and repeated measures. In healthcare research, mental health outcomes often exhibit non-normal distributions, and GLMMs can handle various distributional forms. Given the longitudinal nature of the study (collecting data over time from the same individuals), GLMMs are valuable. Because the response variables (depression and anxiety) were categorized as dichotomous variables, the binomial logistic approach with the logit link function model was applied. Occupation, age, working experience, workplace, education, direct contact with COVID-19 patients in T0, sex, working hours, and self-esteem were used as explanatory variables. Explanatory variables were selected by evaluating the *p*-value and conducting filter methods—evaluating each variable’s importance based on statistical measures such as correlation coefficients (for example, rank biserial correlation). To build the regression model, both forward and backward stepwise regression methods were used. All possible models, including the null model and interactions, were calculated. To select the best model, the Akaike Information Criterion (AIC) was used. The variables included in the final model for depression are workplace, education, direct contact with COVID_19 patients and self-esteem; for anxiety- workplace, sex, direct contact with COVID-19 patients, age and self-esteem. The statistical data analysis was conducted using the Jamovi program (v.2.3.28) with the “gamlj” module [28,29,30].

## 3. Results

In T0, 322 and 352 participants were included in the data analysis for depression and anxiety, respectively. The parameters of the baseline and follow-up samples are seen in Table 1 and Table 2.

The incidence density of depression and anxiety was calculated (Table 3 and Table 4). Analysis of the incidence density highlights the association of self-esteem with the incidence of depression—participants with medium and low self-esteem reported higher rates of depression compared to participants with high self-esteem during the COVID-19 pandemic in Latvia (*p* < 0.001; *p* < 0.001). HCWs over the age of 50 had lower depression incidence rates (*p* = 0.017) than younger HCWs. Working experience of more than 20 years compared to working experience of less than five years is a protective factor for depression (21–30 years versus <5 years (*p* = 0.011); >30 years versus <5 years (*p* = 0.007)) in the study sample. 

Low self-esteem compared to high self-esteem was a significant risk factor for the incidence of anxiety among HCWs during the COVID-19 pandemic in Latvia (*p* < 0.001). Compared to HCWs aged 19–29 years old, HCWs aged over 50 reported lower anxiety incidence rates (*p* = 0.024). 

Other factors did not leave a significant mark on the results of the incidence density analysis. Further GLMM analysis was carried out to control for possible negative confounders.

GLMM was performed to identify the factors associated with the incidence of depression and anxiety among HCWs (Table 5 and Table 6). Participants with medium self-esteem in T0 had a 61% risk of depression, and those with low self-esteem in T0 had an 83% risk of depression (*p* < 0.001; *p* < 0.001). Working at a GP practice was associated with a twice higher risk of depression compared to working at a hospital or SEMS (*p* < 0.001). Participants with higher education had a 29% lower risk of depression (*p* = 0.007). Those who had direct contact with COVID-19 patients had a 31% higher risk of depression (*p* = 0.005).

Participants with medium self-esteem at T0 had a 44% risk of anxiety, and those with low self-esteem at T0 had a 76% risk of anxiety (*p* < 0.001; *p* < 0.001). Working at a GP practice was associated with a twice higher risk of anxiety compared to working at a hospital and SEMS (*p* < 0.001), but working in Stomatology was associated with a 44% lower risk of anxiety compared to working at a hospital and SEMS (*p* = 0.40). Males had lower rates of anxiety incidence, but the difference was not statistically significant (*p* = 0.100). Participants who reported direct contact with COVID-19 patients had a higher risk of depression, but this was not statistically significant (*p* = 0.107). Every year of age reduced anxiety incidence rates by 1% (*p* = 0.029). 

## 4. Discussion

To our knowledge, this is the first study that measures the mental health of HCWs at seven different points in time during the COVID-19 pandemic. This study observes HCWs who did not have symptoms of depression and anxiety at the start of the COVID-19 pandemic. The highest prevalence of depression and anxiety was reported in T3—early 2021—after the second wave of COVID-19 in Latvia, which was characterized by higher mortality rates compared to other countries in the European Union [15]. Different dynamics of mental health outcomes are reported. A study in Spain with a relatively small population of 141 participants conducted via face-to-face interviews shows an increase in depression and the state anxiety level in the follow-up conducted six months later [31]. However, a study from New York State (USA) with a follow-up time of one year later and a study from Italy with a follow-up performed after six months showed a significant decrease in mental health problems during the first year of the pandemic; the follow-up was carried out when COVID-19 infection rates were lower, and the mental health of HCWs had improved [13,32]. A study from Italy with measurements taken at three different points in time shows a slight increase in stress, depression, state anger, and emotional exhaustion over one year. The authors explain the dynamics of the symptoms in association with the waves of the COVID-19 pandemic—the first data were collected after the end of the first lockdown, and the first follow-up was conducted during the second wave of infection when Italy was in its second lockdown, and the results of the second follow-up show the importance of moral distress and compassion fatigue during the pandemic [4].

During the COVID-19 pandemic, anxiety was more prevalent than depression. In a recent meta-analysis, the pooled prevalence of depression is 33–36%, and the pooled prevalence of anxiety is 41–47% [1,2]. In Latvia, it was the opposite: depression rates were higher than anxiety rates among HCWs [16]. The frequent follow-up evaluation in our study allowed us to observe incidence cases of depression and anxiety and to analyze how factors evaluated at the beginning of the COVID-19 pandemic could be associated with poor mental health outcomes among HCWs during the COVID-19 pandemic in Latvia.

Association of self-esteem with the incidence of depression and anxiety.

To our knowledge, this is the first longitudinal study of mental health outcomes in relation to baseline self-esteem among HCWs during a pandemic. Our study shows that self-esteem is a more important factor associated with the incidence of depression and anxiety than occupational factors. Low self-esteem at the beginning of the pandemic increased the risk of depression by 87% and the risk of anxiety by 76%. We can see similarities with the results of the meta-analysis by Sowislo and colleagues in our study: the risk of depression is higher than the risk of anxiety among HCWs with low self-esteem [12]. 

A study from Croatia reports an association between the self-esteem of HCWs and organizational problems in healthcare settings [33]. Because the healthcare system in Latvia has been underfunded for many years, human resources also suffer [15]. The median age of HCWs has increased in Latvia. Because of a shortage of doctors and nurses, medical students were recruited to work with COVID-19 patients [17]. HCWs are already recognized as a risk group for lower self-esteem at the time of starting their medical studies. Nursing students have lower self-esteem than business administration and finance students. The self-esteem of business administration and finance students increases with each study year, and no such correlation is observed in the population of nursing students [34]. Self-esteem is important in the professional life of future HCWs after their studies. Primary health professionals with good leadership work more efficiently and are satisfied with their work, resulting in better patient care [35]. 

Association of sociodemographic factors with the incidence of depression and anxiety.

The results of several cross-sectional studies show differences among the sexes regarding mental health disorders: women experienced anxiety and depression more frequently than men during the COVID-19 pandemic [3,6]. The results of two longitudinal studies show higher anxiety rates among women but no significant differences in depression [13,36]. A longitudinal study in Italy shows higher depression and anxiety rates in women, but the difference is not statistically significant [5]. A study from Canada also shows no differences between the sexes in terms of depression and anxiety [37]. Our study records similar results—sex is not a statistically significant factor for depression and anxiety among HCWs. Sex is an important co-factor for the incidence of anxiety, but it is not statistically significant.

The effect of age on depression and anxiety among HCWs is inconsistent. Some studies show that age is not an important factor for anxiety and depression [4,5,37]. However, another study found an association between younger age and depression among HCWs [36]. The results of our study show that the risk of depression is not associated with age, but the risk of anxiety decreases by 1% every year among HCWs. Incidence density analysis revealed an association between more working experience and lower depression rates, but the effect diminished after performing GLMM.

Association of occupational factors with the incidence of depression and anxiety.

The profession has been a widely studied factor in mental health issues among HCWs. Being a nurse is a well-known risk factor for depression and anxiety during the COVID-19 pandemic [3,6]. In the meta-analysis of Dragioti and colleagues, working as a doctor is associated with stress and PTSD [6]. However, another meta-analysis shows that doctors, nurses, and older HCWs experience depression and anxiety more often [2]. In our study, there is no difference in the incidence of depression and anxiety among professions; however, we identified the importance of education. In HCWs with a higher level of education, the risk of depression is 29% lower than in those with a lower level of education. Some studies have found that nurses have higher rates of anxiety than physicians [4,5] and that nurses and healthcare assistants face a higher risk of anxiety and depression [13].

We find two possible explanations for why profession is not significant for the incidence of depression and anxiety in our sample, but education is. Firstly, medical education and working requirements in Latvia include undergraduate and post-graduate studies for nurses and physician assistants. Secondly, the stratification group ‘Other’ includes a wide range of professions with different levels of education: physiotherapists, dental technicians, medical students, etc.

Several study results show that working in a hospital is associated with a higher risk of mental health problems among HCWs. The results of the meta-analysis published in 2022 show similar results to the results published in 2023—the prevalence of depression is 28.4% and 28.5%, respectively; the prevalence of anxiety is 29.9% and 28.7%, respectively [3,6].

In our study, working at a GP practice is associated with twice the risk of developing depression or anxiety. Our data are consistent with a study from France—in follow-up questionnaires, burnout increases among GPs [38]. GPs in Latvia work mainly in private practices with small teams and less social support, and they faced new regulations and duties during the COVID-19 pandemic, for example, remote consultations (by phone) and management of the COVID-19 vaccination process [17].

In our study, working hours were not a significant factor in the incidence of anxiety or depression. A similar finding came from a study conducted in New York, USA. The study reports longer working hours among HCWs but no association with mental health outcomes [32]. This could imply that working conditions and workload during working hours are more important than working hours per se. For example, in our study, we saw that GP-practice-based HCWs worked normal working hours, but their depression and anxiety rates were the highest. A study from Italy had similar results: depression and anxiety rates are positively associated with working in an environment with an increased intensity of care [13]. Qualitative studies are needed to explore the burden of working hours.

Working conditions are important for mental health outcomes during the COVID-19 pandemic. Working on the frontlines in high-risk COVID-19 units and intensive/emergency care units is associated with anxiety and depression [3,6,13,36]. However, some studies did not find working in COVID-19 units significant for depression and anxiety [4,5]. Other studies show that direct contact with COVID-19 patients is associated with depression and anxiety [3]. In our study, we examined direct contact with COVID-19 patients. After performing GLMM, it was significantly associated with a 30% higher risk of depression during the COVID-19 pandemic. Direct contact with COVID-19 patients is an essential co-factor for the incidence of anxiety, but it is not statistically significant.

This study has some limitations. After analyzing the data and research design in detail, we concluded that it is impossible to calculate the average time it takes for depression or anxiety to occur. One of the main reasons is the size of the sample and the frequency of the measurements. However, the measurement frequency helped to detect incidences of depression and anxiety more accurately. Dropout and the length of the questionnaire could introduce a sampling bias. There is a higher possibility that HCWs who suffer from negative mental health outcomes feel an additional burden to fill out the questionnaire. Due to the heterogeneity of participant recruitment and a non-probability sampling approach, the response rate cannot be determined. Because of the sampling approach, there are limitations to generalizing the results to all HCWs in Latvia. Still, in comparison with studies from larger countries, our sample size is comparable and representable—all areas of the healthcare system from regions of Latvia were included and represented to provide a generalized impression of mental health status during the COVID-19 pandemic in Latvia. This study focused on depression and anxiety because most of the research conducted during the COVID-19 pandemic uses the same scales that are validated in Latvia, and the results can be comparable with the results of similar studies. However, adding more specific mental health outcomes such as PTSD, insomnia, and stress would provide more information on mental health outcomes among HCWs during the COVID-19 pandemic. There is a potential interaction between depression and anxiety, but it was not analyzed due to sample size limitations.

Working hours and workplace do not reflect the real workload—the workload needs to be explored via a more detailed questionnaire. This study highlights the importance of self-esteem in the incidence of depression and anxiety. It addresses the need to explore potential co-founding factors, such as relationship status and satisfaction with it, coping strategies, and personality characteristics, in our next publication. Our study was part of a web-based longitudinal observational study to assess association, not causality. Controlled longitudinal studies are needed to confirm causality.

The practical implications of our study are based on the importance of both self-esteem and occupational factors in association with new cases of depression and anxiety among HCWs during the COVID-19 pandemic in Latvia. A study from Canada reports an increase in outpatient visits from a physician to another physician related to mental health or substance use during the COVID-19 pandemic [39]. Regular screening for self-esteem, depression, and anxiety, especially among HCWs in high-risk units, with further psychiatric or psychotherapeutic approaches, could be beneficial for HCWs in Latvia. 

Working in GP practices is one of the main occupational factors associated with the incidence of depression and anxiety. Reduced workloads and less paperwork from the government would be beneficial. Balint groups are approved to protect HCWs—especially GPs—from burnout [40,41,42]. Government-funded Balint group programs could be useful in the prevention of poor mental health outcomes.

## 5. Conclusions

HCWs with low self-esteem were 83% more likely to experience depression and 76% more likely to experience anxiety compared to those with high self-esteem. Working in GP practices is associated with double the risk of the incidence of depression and anxiety compared to working at hospitals and SEMS. A 31% increase in the odds of depression is observed among HCWs with direct contact with COVID-19 patients compared to those without. Undergraduate education is associated with the incidence of depression. The risk of anxiety decreases by 1% every year in the life of an HCW.

Our study makes apparent the importance of personality factors for HCWs in today’s healthcare system. The organizational and government levels need to search for possibilities to facilitate the mental health of HCWs to ensure better quality healthcare, such as reduced workload and paperwork during working hours and access to psychiatrists, psychotherapists, and Balint groups.

More research based on the association of HCW personality factors with mental health outcomes is needed. Controlled longitudinal studies are needed to confirm the causality of self-esteem and mental health outcomes among HCWs.

## Figures and Tables

**Table 1:** Descriptive characteristics for baseline and follow-up samples for the analysis of depression symptoms.

	T0 (*n* = 322), *n* (%)	T1 (*n* = 273), *n* (%)	T2 (*n* = 265), *n* (%)	T3 (*n* = 224), *n* (%)	T4 (*n* = 201), *n* (%)	T5 (*n* = 206), *n* (%)	T6 (*n* = 166), *n* (%)
Sex							
Women	278 (87.1)	237 (87.1)	230 (87.5)	202 (90.6)	179 (89.5)	184 (90.2)	150 (90.9)
Men	41 (12.7)	35 (12.9)	33 (12.5)	21 (9.4)	21 (10.5)	20 (9.8)	15 (9.1)
Age							
19–29	74 (23.1)	71 (26.0)	64 (24.2)	48 (21.5)	42 (21.0)	41 (20.1)	29 (17.7)
30–39	66 (20.6)	58 (21.2)	60 (22.6)	49 (22.0)	47 (23.5)	48 (23.5)	34 (20.7)
40–49	57 (17.8)	50 (18.3)	45 (17.0)	45 (20.2)	39 (19.5)	41 (20.1)	36 (22.0)
50+	123 (38.4)	94 (34.3)	96 (36.2)	81 (36.3)	72 (36.0)	74 (36.3)	65 (39.6)
Education							
Undergraduate	55 (17.1)	46 (16.8)	42 (15.8)	33 (14.7)	24 (11.9)	26 (12.6)	18 (10.8)
Post-graduate	267 (82.9)	227 (83.2)	223 (84.2)	191 (85.3)	177 (88.1)	180 (87.4)	148 (89.2)
Occupation							
Physician	156 (48.4)	132 (48.4)	134 (50.6)	115 (51.3)	104 (51.7)	103 (50.0)	90 (54.2)
Nurse/physician assistant	125 (38.8)	106 (38.8)	96 (36.2)	86 (38.4)	80 (39.8)	85 (41.3)	65 (39.2)
Other	41 (12.7)	35 (12.8)	35 (13.2)	23 (10.3)	17 (8.5)	18 (8.7)	11 (6.6)
Workplace							
Hospital and SEMS	223 (69.3)	187 (68.5)	180 (67.9)	150 (67.0)	132 (65.7)	133 (64.6)	103 (62.0)
GP	79 (24.5)	70 (25.6)	68 (25.7)	57 (25.4)	52 (25.9)	59 (28.6)	51 (30.7)
Stomatology	20 (6.2)	16 (5.9)	17 (6.4)	17 (7.6)	17 (8.5)	14 (6.8)	12 (7.2)
Working experience							
<5	59 (18.7)	57 (21.3)	49 (18.9)	35 (15.8)	27 (13.6)	29 (14.3)	16 (9.8)
5–10	66 (20.9)	57 (21.3)	61 (23.6)	47 (21.2)	44 (22.2)	45 (22.2)	34 (20.7)
11–20	55 (17.4)	49 (18.3)	45 (17.4)	42 (18.9)	40 (20.2)	40 (19.7)	30 (18.3)
21–30	53 (16.8)	45 (16.8)	47 (18.1)	40 (18.0)	38 (19.2)	38 (18.7)	35 (21.3)
>30	83 (26.3)	60 (22.4)	57 (22.0)	58 (26.1)	49 (24.7)	51 (25.1)	49 (29.9)
Working hours							
<40	75 (23.5)	52 (19.2)	44 (16.7)	29 (13.1)	26 (13.1)	36 (17.6)	22 (13.4)
40–48	161 (50.5)	116 (42.8)	100 (38.0)	87 (39.2)	89 (44.7)	74 (36.1)	67 (40.9)
>48	83 (26.0)	103 (38.0)	119 (45.2)	106 (47.7)	84 (42.2)	95 (46.3)	75 (45.7)
Direct contact with COVID-19 patients	175 (54.5)	117 (42.9)	163 (61.5)	160 (71.4)	133 (66.2)	145 (70.4)	120 (72.3)
Self-esteem							
Low	22 (6.9)	27 (9.9)	24 (9.1)	30 (13.5)	24 (11.9)	32 (15.5)	24 (14.5)
Medium	44 (13.8)	40 (14.7)	54 (20.4)	42 (18.8)	36 (17.9)	37 (18.0)	26 (15.7)
High	254 (79.4)	206 (75.5)	187 (70.6)	151 (67.7)	141 (70.1)	137 (66.5)	116 (69.9)
Anxiety	20 (6.2)	24 (8.8)	39 (14.7)	41 (18.3)	24 (11.9)	45 (21.8)	35 (21.1)
Depression		30 (11.0)	49 (18.5)	68 (30.4)	43 (21.4)	51 (24.8)	40 (24.1)

**Table 2:** Descriptive characteristics for baseline and follow-up samples for the analysis of anxiety symptoms.

	T0 (*n* = 352), *n* (%)	T1 (*n* = 298), *n* (%)	T2 (*n* = 286), *n* (%)	T3 (*n* = 242), *n* (%)	T4 (*n* = 216), *n* (%)	T5 (*n* = 223), *n* (%)	T6 (*n* = 182), *n* (%)
Sex							
Women	301 (86.2)	256 (86.2)	246 (86.6)	218 (90.5)	190 (88.4)	200 (90.5)	164 (90.6)
Men	48 (13.8)	41 (13.8)	38 (13.4)	23 (9.5)	25 (11.6)	21 (9.5)	17 (9.4)
Age							
19–29	82 (23.5)	80 (26.9)	69 (24.2)	51 (21.3)	48 (22.4)	45 (20.5)	36 (20.1)
30–39	70 (20.1)	61 (20.5)	61 (21.4)	50 (20.8)	47 (22.0)	48 (21.8)	33 (18.4)
40–49	63 (18.1)	54 (18.2)	50 (17.5)	48 (20.0)	41 (19.2)	45 (20.5)	39 (21.8)
50+	134 (38.4)	102 (34.3)	105 (36.8)	91 (37.9)	78 (36.4)	82 (37.3)	71 (39.7)
Education							
Undergraduate	57 (16.2)	48 (16.1)	44 (15.4)	34 (14.0)	25 (11.6)	27 (12.1)	20 (11.0)
Post-graduate	295 (83.8)	250 (83.9)	242 (84.6)	208 (86.0)	191 (88.4)	196 (87.9)	162 (89.0)
Occupation							
Physician	172 (48.9)	148 (49.7)	146 (51.0)	128 (52.9)	113 (52.3)	115 (51.6)	100 (54.9)
Nurse/physician assistant	139 (39.5)	115 (38.6)	105 (36.7)	92 (38.0)	86 (39.8)	90 (40.4)	69 (37.9)
Other	41 (11.6)	35 (11.7)	35 (12.2)	22 (9.1)	17 (7.9)	18 (8.1)	13 (7.1)
Workplace							
Hospital and SEMS	241 (68.5)	202 (67.8)	191 (66.8)	159 (65.7)	140 (64.8)	139 (62.3)	111 (61.0)
GP	90 (25.6)	79 (26.5)	77 (26.9)	66 (27.3)	58 (26.9)	69 (30.9)	58 (31.9)
Stomatology	21 (6.0)	17 (5.7)	18 (6.3)	17 (7.0)	18 (8.3)	15 (6.7)	13 (7.1)
Working experience							
<5	66 (19.0)	65 (22.2)	52 (18.5)	38 (15.8)	33 (15.4)	33 (14.9)	22 (12.2)
5–10	71 (20.5)	60 (20.5)	66 (23.5)	48 (20.0)	45 (21.0)	45 (20.4)	34 (18.8)
11–20	62 (17.9)	54 (18.4)	50 (17.8)	46 (19.2)	41 (19.2)	44 (19.9)	34 (18.8)
21–30	55 (15.9)	46 (15.7)	48 (17.1)	40 (16.7)	40 (18.7)	39 (17.6)	35 (19.3)
>30	93 (26.8)	68 (23.2)	65 (23.1)	68 (28.3)	55 (25.7)	60 (27.1)	56 (30.9)
Working hours							
<40	84 (24.1)	61 (20.6)	49 (17.3)	35 (14.5)	31 (14.5)	42 (18.9)	25 (13.9)
40–48	166 (47.6)	118 (39.9)	102 (35.9)	92 (38.2)	90 (42.1)	76 (34.2)	74 (41.1)
>48	99 (28.4)	117 (39.5)	133 (46.8)	114 (47.3)	93 (43.5)	104 (46.8)	81 (45.0)
Direct contact with COVID-19 patients	191 (54.4)	131 (44.0)	174 (60.8)	173 (71.5)	143 (66.2)	153 (68.6)	135 (74.2)
Self-esteem							
Low	34 (9.7)	39 (13.1)	34 (11.9)	38 (15.8)	29 (13.4)	42 (18.8)	33 (18.1)
Medium	51 (14.6)	42 (14.1)	57 (19.9)	46 (19.1)	43 (19.9)	37 (16.6)	32 (17.6)
High	264 (75.6)	217 (72.8)	195 (68.2)	157 (65.1)	144 (66.7)	144 (64.6)	117 (64.3)
Anxiety		29 (9.7)	43 (15.0)	45 (18.6)	33 (15.3)	53 (23.8)	45 (24.7)
Depression	49 (13.9)	50 (16.8)	64 (22.4)	85 (35.1)	55 (25.5)	70 (31.4)	54 (29.7)

**Table 3:** Incidence density of depression symptoms.

	Total (*n*)	Depression (*n* (%))	Person-Months	Incidence Density (ID)	ID 95% CI	*p*-Values
Sex						
Women	278	111 (39.9)	3326	0.03	0.03 to 0.04	0.538
Men	41	16 (39.0)	409	0.04	0.02 to 0.06	
Occupation						
Physician *	156	67 (42.9)	1909	0.04	0.03 to 0.04	
Nurse/physician assistant	125	46 (36.8)	1505	0.03	0.02 to 0.04	0.479
Other	41	15 (36.6)	359	0.04	0.02 to 0.07	0.541
Self-esteem						
Low	22	14 (63.6)	196	0.07	0.04 to 0.12	<0.001
Medium	44	28 (63.6)	420	0.07	0.04 to 0.10	<0.001
High *	254	85 (33.5)	3132	0.03	0.02 to 0.03	
Age						
19–29 *	74	33 (44.6)	720	0.05	0.03 to 0.06	
30–39	66	31 (47.0)	782	0.04	0.03 to 0.06	0.561
40–49	57	22 (38.6)	684	0.03	0.02 to 0.05	0.196
50+	123	41 (33.3)	1552	0.03	0.02 to 0.04	0.017
Working experience						
<5 *	59	27 (45.8)	522	0.05	0.03 to 0.08	
5–10	66	31 (47.0)	725	0.04	0.03 to 0.06	0.469
11–20	55	23 (41.8)	657	0.04	0.02 to 0.05	0.166
21–30	53	17 (32.1)	711	0.02	0.01 to 0.04	0.011
>30	83	28 (33.7)	1109	0.03	0.02 to 0.04	0.007
Workplace						
Hospital and SEMS	223	82 (36.8)	2575	0.03	0.03 to 0.04	0.109
GP *	79	40 (50.6)	923	0.04	0.03 to 0.06	
Stomatology	20	6 (30.0)	275	0.02	0.01 to 0.05	0.110
Education						
Undergraduate	55	18 (32.7)	555	0.03	0.02 to 0.05	0.836
Post-graduate	267	110 (41.2)	3218	0.03	0.03 to 0.04	
Direct contact with COVID-19 in T0						
No	146	58 (39.7)	1812	0.03	0.02 to 0.04	0.503
Yes	175	70 (40.0)	1942	0.04	0.03 to 0.05	
Direct contact with COVID-19 in T0–T6						
No	46	19 (41.3)	507	0.04	0.02 to 0.06	0.641
yes	276	109 (39.5)	3266	0.03	0.03 to 0.04	
Working hours						
<40 *	74	29 (39.2)	853	0.03	0.02 to 0.05	
40–48	114	41 (36.0)	1348	0.03	0.02 to 0.04	0.646
>48	132	57 (43.2)	1547	0.04	0.03 to 0.05	0.724

* Reference.

**Table 4:** Incidence density for anxiety symptoms.

	Total (*n*)	Anxiety (*n* (%))	Person-Months	Incidence Density (ID)	ID 95% CI	*p*-Values
Sex						
Women	301	103 (34.2)	3842	0.03	0.02 to 0.03	0.500
Men	48	16 (33.3)	498	0.03	0.02 to 0.05	
Occupation						
Physician *	172	68 (39.5)	2219	0.03	0.02 to 0.04	
Nurse/physician assistant	139	44 (31.7)	1762	0.02	0.02 to 0.03	0.289
Other	41	8 (19.5)	397	0.02	0.01 to 0.04	0.259
Self-esteem						
Low	34	21 (61.8)	293	0.07	0.04 to 0.11	<0.001
Medium	51	20 (39.2)	569	0.04	0.02 to 0.05	0.061
High *	264	77 (29.2)	3491	0.02	0.02 to 0.03	
Age						
19–29 *	82	30 (36.6)	848	0.04	0.02 to 0.05	
30–39	70	28 (40.0)	875	0.03	0.02 to 0.05	0.703
40–49	63	23 (36.5)	809	0.03	0.02 to 0.04	0.429
50+	134	37 (27.6)	1808	0.02	0.01 to 0.03	0.024
Working experience						
<5 *	66	22 (33.3)	638	0.03	0.02 to 0.05	
5–10	71	28 (39.4)	837	0.03	0.02 to 0.05	0.915
11–20	62	23 (37.1)	789	0.03	0.02 to 0.04	0.573
21–30	55	19 (34.5)	773	0.02	0.02 to 0.04	0.277
>30	93	27 (29.0)	1291	0.02	0.01 to 0.03	0.079
Workplace						
Hospital and SEMS	241	74 (30.7)	2867	0.03	0.02 to 0.03	0.203
GP *	90	40 (44.4)	1208	0.03	0.02 to 0.05	
Stomatology	21	6 (28.6)	303	0.02	0.01 to 0.04	0.235
Education						
Undergraduate	57	16 (28.1)	600	0.03	0.02 to 0.04	0.906
Post-graduate	295	104 (35.3)	3778	0.03	0.02 to 0.03	
Direct contact with COVID-19 in T0						
No	160	55 (34.4)	2175	0.03	0.02 to 0.03	0.427
Yes	191	64 (33.5)	2187	0.03	0.02 to 0.04	
Direct contact with COVID-19 in T0–T6						
No	50	15 (30.0)	604	0.02	0.01 to 0.04	0.681
yes	302	105 (34.8)	3774	0.03	0.02 to 0.03	
Working hours						
<40 *	84	27 (32.1)	1063	0.03	0.02 to 0.04	
40–48	117	38 (32.5)	1437	0.03	0.02 to 0.04	0.873
>48	150	55 (36.7)	1872	0.03	0.02 to 0.04	0.535

* Reference.

**Table 5:** Fixed effects in GLMM to identify factors influencing the occurrence of depression. Data were analyzed by applying the binomial logistic (with logit link function) GLMM, including time as a random effect variable.

Parameter	Category	OR	95% CI of OR	z	*p*
Intercept		0.11	0.53–0.81	−3.96	<0.001
Workplace	GP*–*Hospital and SEMS *	2.04	1.67–2.49	6.99	<0.001
	Stomatology*–*Hospital and SEMS *	0.68	0.43–1.09	−1.58	0.114
Education	Post-graduate*–*Undergraduate *	0.71	0.55–0.91	−2.69	0.007
Direct contact	Yes*–*No *	1.31	1.09–1.57	2.82	0.005
Self-esteem	Moderate*–*Low *	0.39	0.30–0.51	−7.15	<0.001
	High*–*Low *	0.17	0.14–0.22	−15.79	<0.001

* Reference.

**Table 6:** Fixed effects in GLMM to identify factors influencing the occurrence of anxiety. Data were analyzed by applying the binomial logistic (with logit link function) GLMM, including time as a random effect variable.

Parameter	Category	OR	95% CI of OR	z	*p*
Intercept		0.32	0.25–0.40	−9.65	<0.001
Workplace	GP–Hospital *	2.06	1.67–2.55	6.64	<0.001
	Stomatology–Hospital *	0.56	0.32–0.97	−2.05	0.040
Sex	Male*–*Female *	0.76	0.55–1.05	−1.65	0.100
Direct contact	Yes*–*No *	1.18	0.97–1.43	1.61	0.107
Self-esteem	Moderate*–*Low *	0.56	0.43–0.73	−4.31	<0.001
	High*–*Low *	0.24	0.20–0.30	−12.38	<0.001
Age		0.99	0.98–0.99	−2.19	0.029

* Reference.

## Data Availability

Data are unavailable due to privacy and ethical restrictions.
